# Ethnic and socio-economic disparities in oral health outcomes and quality of life among Sri Lankan preschoolers: a cross-sectional study

**DOI:** 10.1186/1475-9276-12-89

**Published:** 2013-11-14

**Authors:** Vajira Nanayakkara, André Renzaho, Brian Oldenburg, Lilani Ekanayake

**Affiliations:** 1 Global Health and Society Unit, Department of Epidemiology and Preventive Medicine, Monash University, Level 3, Burnet Building, 89 Commercial Rd, Melbourne, Victoria 3004, Australia; 2 Ministry of Healthcare and Nutrition, Colombo, Sri Lanka; 3 Global Health and Society Unit, Monash University; Burnet Institute, Level 3, Burnet Building, 89 Commercial Rd, Melbourne, Victoria 3004, Australia; 4 Department of Community Dental Health, Faculty of Dental Sciences, University of Peradeniya, Peradeniya, Sri Lanka

**Keywords:** Oral health, Dental caries, Bleeding gums, Quality of life, Socio-economic disparities, Preschoolers, Sri Lanka

## Abstract

**Introduction:**

The distribution and severity of dental caries among preschool children vary according to the socio-economic and ethnic differences within and between countries. Understanding socio-economic influences on child oral health could inform early interventions to reduce the oral health burden throughout the life-cycle. The aim of this study is to examine the socio-economic and ethnic influences on oral health among preschoolers in Kegalle, Sri Lanka.

**Methods:**

The study involved 784 children aged between 48–72 months recruited from 84 pre-schools in the Kegalle district in Sri Lanka. Cross-sectional data were collected by means of an oral examination of the children and a self-administered questionnaire to their parents/caregivers. The Early Childhood Oral Health Impact Scale (ECOHIS) was used to assess Oral Health related Quality of Life (OHQoL). Univariate and multivariate models of Poisson regression were used to investigate the associations between the variables.

**Results:**

Compared to children whose fathers had tertiary education, those whose fathers did not study beyond grade 5, had more caries measured in terms of decayed, missing and filled surfaces (dmfs) (IRR = 2.30; 95% CI: 1.30, 4.06; p < 0.01) and experienced poor OHQoL at child (IRR = 2.52; 95% CI: 1.20, 5.31; p < 0.05) and family (IRR = 1.59; 95% CI: 1.11, 2.27; p < 0.05) levels. However, lower educational attainment among mothers was associated with better OHQoL among children. Compared to the Sinhalese ethnic group, Tamils had more gingival bleeding (bleeding surfaces) (IRR = 3.04; 95% CI: 1.92, 4.81; p < 0.001) and poor OHQoL at child level (IRR = 2.07; 95% CI: 1.19, 3.60; p < 0.01), whereas Muslims had poor OHQoL at family level (IRR = 1.42; 95% CI: 1.10, 1.84; p < 0.01). Children of low-income families had more gum bleeding (IRR = 1.00; 95% CI: 0.99, 1.00; p < 0.05) compared to children of high-income families.

**Conclusions:**

Socio-economic and ethnic differences in oral health outcomes exist among this population of preschoolers. Interventions targeting children of fathers with low educational levels and ethnic minority groups are required to reduce inequalities in oral health in Sri Lanka and other similar countries.

## Introduction

Dental caries and periodontal disease account for a considerable proportion of the global burden of oral diseases [1], thus causing an enormous loss of school and working hours. Research has demonstrated that the oral health of the child makes an important contribution to the wellbeing of both the child and the family [2]. However, since poor dental behaviours established at preschool age may be difficult to modify, preschool age is a critical period in oral health [3]. Some intervention studies have been carried out to investigate the effectiveness of measures taken to prevent caries in young children. Visiting mothers with infants by trained dental health educators at the time of eruption of first deciduous teeth or soon after was demonstrated to be effective in preventing nursing caries [4]. However, Rayner has shown that preventive programs have only a little effect on the uptake of dental care in nursery and young primary school children, although the provision of health education to their parents may be effective in improving the oral hygiene of children [5]. The distribution and severity of oral diseases vary within and between countries depending on socio-economic status and ethnic differences [6-10]. In several Asian and African countries the level of caries is higher in the primary dentition than in the permanent dentition in children [11,12], and poor oral health is unequally distributed in these countries, with those from socially-disadvantaged communities among the most affected [13]. For example, in Sri Lanka, the prevalence of dental caries in primary dentition of 5 years old children is 65% [12], while the national figure for the prevalence of bleeding gums for the same age group is 46.3% [14]. Similarly high caries levels have been reported in some other countries in Asia and Africa [15-17]. However, in developed countries the prevalence of dental diseases is not as high as in developing countries. For example, in the USA it has been demonstrated that the prevalence of dental caries among 12–60 month old children is 28% [18], and in Brisbane, Australia among 4–6 year olds caries prevalence is 33.7% [19].

Oral diseases are of multi-factorial etiology. The important role of socio-behavioural and environmental factors in oral health and oral diseases has been shown in many different epidemiological surveys [1]. Fisher-Owens and colleagues [20] have shown the importance of assessing the influences of socio-behavioural and environmental factors on oral health. Moreover, the influence of these factors on OHQoL in childhood and old age differs across settings [21-23]. In Sri Lanka, some studies have focused on the influence of demographic and socio-economic factors on the oral health of children [24-27]. These studies have looked at a broad range of factors affecting caries prevalence, the utilization of dental services and perceptions of oral health among different age groups, including preschoolers. Findings from these studies suggest that birth rank, family size, gender and income of the family are important determinants of various aspects of oral health. However, evidence of the effect of demographic and socio-economic factors on OHQoL of the pre-school age is lacking.

The aim of the present study was to assess the effect of selected socio-demographic factors on dental caries, gingival bleeding and OHQoL in preschool children in the Kegalle district in Sri Lanka. It was hypothesized that there are ethnic differentials in oral health and OHQoL and that children from disadvantaged families have poorer oral health outcomes, as measured by dental caries, gingival bleeding and OHQoL. Ethnic and socio-economic differences in oral health outcomes and OHQoL are reported and the possible explanations are discussed.

## Methods

This study was carried out among 48–72 month-old preschool children in the Kegalle district of Sri Lanka, which has an agricultural economy with a population of almost 780,000 people [28]. The study used a cross-sectional design and a formula for estimating a population proportion with absolute precision was used to calculate the sample size. Considering 65% as the prevalence estimate of caries in 6 year olds [14], at a 95% confidence interval the calculated minimum sample size was 350. Making allowances for a 2% design effect and a 15% non-response rate, the required sample size was 805. Initially 838 subjects were enrolled in the study, which exceeded the required sample size. None of the parents refused to take part in the study and all answered the questionnaires. Furthermore, no handicapped children were encountered, who would have been excluded. However, the final analysis included only 784 subjects who completed oral examinations, giving a response rate of 94%.

The study sample was selected using the cluster sampling technique. A preschool was considered as a cluster. Since more clusters with fewer individuals would reduce the cluster effect [29], it was decided to include 84 clusters with ten children in each. For administrative purposes, Kegalle District is divided in to 11 Divisional Secretariat Divisions (DSDs). Based on the preschool population in each DSD, the number of clusters to select from each DSD was determined according to the probability proportionate to size technique. The minimum number of clusters selected in a DSD was 4, and the maximum was 14, according to their preschool population.

According to the following steps the required number of preschools (clusters) was identified in each DSD. The main commercial location of the area was identified. The closest preschool was selected as the first cluster from that area. The preschool closest to the first in a randomly selected direction was selected as the second cluster. This method was followed until the required number of clusters was identified from each DSD. If a selected preschool had more than ten eligible children, ten children were selected by drawing lots. If a selected preschool did not have ten eligible children, the remaining number was selected from the next nearest preschool.

OHQoL was assessed by the Early Childhood Oral Health Impact Scale (ECOHIS) [2], which measures the impact of oral health problems of the preschool child on the quality of life of the child as well as his/her family. ECOHIS includes nine items related to the child and four items to the family. Calculation of ECOHIS scores was done according to the method suggested by the authors of the scale. A “do not know” response was considered as a missing response. Cases with more than 2 missing responses in Section 1 the child, and more than one missing response in Section 2 the family, were excluded from the relevant sections during the analysis. If any respondent had only 1 or 2 missing responses for the child section and 1 missing response for the family section, for those responses, a value was given by averaging the score of the other questions answered.

Guidance of a communication specialist was obtained in order to refine the language of ECOHIS for better clarity and comprehension. The face validity and content validity of the translated questionnaire were assessed by a consultant in Community Medicine. Consensual validity of the questionnaire was ensured by considering the comments of two experts in public health. The construct validity of the questionnaire was assessed by administering it to a group of mothers of preschool children and by determining the associations between the quality of life scores in the child and family sections and the parent’s/caregiver’s rating of the child’s oral health. The internal consistency of the questionnaire was assessed using Cronbach’s alpha. The Cronbach’s alpha for the child section was 0.93 and for the family section 0.91, indicating good internal consistency.

ECOHIS was validated in the Sinhalese language. However each time the questionnaire was applied to a member of the Tamil ethnic group, a Tamil translation of the questionnaire prepared by an expert in both languages was used to explain the questions to the respondent by the preschool teacher who served as an interpreter.

Data were collected by means of self-administered questionnaires to each parent/caregiver and an oral examination of the child. Assistance in answering the questionnaires was provided by a trained person for those who had difficulty in understanding the contents. Dental caries and gingival bleeding were assessed by the first author under natural daylight while the child was seated on a chair. The examiner was trained and calibrated with a specialist in paediatric dentistry on the recording of caries and gingival bleeding. Caries was recorded as the dmfs score according to the criteria recommended by Drury et al. [30]; non-cavitated lesions, cavitated lesions, missing due to caries and filled. Gingival bleeding was assessed by running a periodontal probe along the cervical margins on 4 surfaces of 6 teeth; the distal, buccal/labial and lingual/palatal surfaces of 55, 61, 63, 75, 81 and 83, as suggested by Ramfjord [31], and the number of surfaces with bleeding was determined. Intra – examiner agreement associated with caries detection and gingival bleeding as determined by the Kappa statistic was 0.89 and 0.85 respectively, representing excellent agreement [29].

The questionnaire was pre-tested on a group of 20 mothers of preschool children who were not included in the main study. Based on the findings of the pre-test, certain questions were rephrased for better clarity. A pilot study was carried out with another group of 20 mothers and their children who were from the same area but excluded from the main study. This included the administration of the questionnaires as well as examinations of the children. The time required for administering the questionnaires and for the physical and oral examinations of a child was determined.

Stata Version 11 (Stata Corporation, College Station, TX, USA) was used for statistical analysis. Family income was reported in Sri Lankan Rupees. However, for the purpose of analysis, income and age were entered as continuous variables. Educational level of mother and father was reported in three levels, which were derived from the categorization used in the Demographic and Health Survey Sri Lanka 2006/7 [32]. Since very few respondents had no schooling, both “no education” and “primary education” were categorised as “≤ 5 grade”. “Secondary education” and “passed GCE O/L (up to GCE A/L)” were included in the category “6-12 grade”. Those who had attained more than GCE A/L (higher) were categorized in the “ >12 grade” (diploma/degree or tertiary education).

Since our dependent variables were count data, adjusted robust Poisson regression bivariate and multivariate models were performed to obtain the incidence rate ratio (and its 95% confidence intervals) and to determine factors associated with poor oral health (dental caries and gingival bleeding) and OHQoL. We used the vce (robust) option in STATA to obtain robust standard errors for the parameter estimates and to control for mild violation of underlying assumptions [33]. Significance was retained at P < 0.05.

The study was approved by the Ethics Review Committee, Faculty of Medicine, University of Colombo, Sri Lanka. Written informed consent of the parents/caregivers was also obtained.

## Results

The demographic characteristics of the sample and oral health outcomes are presented in Tables 1 and 2 respectively. A total of 838 parent–child pairs were invited to take part in the study, but only 784 children took part in the oral examinations, giving a response rate of 94%.

**Table 1:** Distribution of sample according to selected socio-demographic variables

**Variable**	**n**	**%**
Total	784	100
Age		
48-60 months	466	59.4
61-72 months	318	40.6
Mean age (SD)	57.30	(6.24)
Gender		
Boys	383	48.9
Girls	401	51.1
Ethnicity		
Sinhala	704	89.8
Muslim	62	7.9
Tamil	18	2.3
Family income (Sri Lankan Rupees)		
<10,000	416	53.6
10,001-20,000	221	28.5
20,001 and above	138	17.8
non-response	9	
Mean income (SD)	14967.03	(10449.24)
Education level of mother		
≤ grade 5	35	4.5
grade 6-12	636	81.5
> grade 12 (degree/diploma)	109	14.0
non-response	4	
Education level of/ father		
≤ grade 5	52	6.7
grade 6-12	656	84.1
> grade 12 (degree/diploma)	72	9.2
non-response	4	

Non-respondents were excluded when calculating the percentages.

**Table 2:** Distribution of sample according to oral Health outcomes and oral Health-related Quality of Life

**Variable**	**n**	**%**
Total	784	100
Dental caries		
caries present	564	71.9
caries absent	220	28.1
Mean (SD) dmfs	6.4 (8.5)	
Gingival bleeding		
bleeding present	369	47.1
bleeding absent	415	52.9
Mean (SD) bleeding surfaces	1.1 (1.5)	
Oral health-related quality of life		
children affected	332	42.5
children not affected	449	57.5
non-response	3	
Mean (SD) ECOHIS child score	2.2 (3.6)	
families affected	164	21.0
families not affected	617	79.0
non-response	3	
Mean (SD) ECOHIS family score	0.6 (1.5)	

Non-respondents were excluded when calculating the percentages.

In the unadjusted analyses, high caries levels were associated with Tamil ethnicity, lower household income and low educational level of father and mother, while higher gingival bleeding levels were associated with older age, being a boy, belonging to Muslim and Tamil ethnic groups, low household income level and grade 5 or lower level of education of mother (Table 3). Poor OHQoL among children was associated with older age, Muslim and Tamil ethnicities and lower levels of father’s and mother’s education. Lower OHQoL of family was associated with Muslim ethnicity, mother’s education (grade 6–12) and father’s education (grade 6–12) (Table 4).

**Table 3:** Associations between selected socio-demographic variables and oral health outcomes (caries and bleeding gums)

**Variables**	**Caries ( dmfs)**				**Bleeding gums (bleeding surfaces)**			
	**Unadjusted coefficient (SE)**	**IRR (95% CI)**	**Adjusted coefficient (SE)**	**IRR (95% CI)**	**Unadjusted coefficient (SE)**	**IRR (95% CI)**	**Adjusted coefficient (SE)**	**IRR (95% CI)**
Age	0.01 (0.01)	1.01 (0.99,1.03)	−0.00 (0.01)	0.99 (0.98,1.01)	0.02^*^ (0.01)	1.02^*^ (1.00,1.04)	0.02 (0.01)	1.02 (1.00,1.04)
Gender								
Boys	0.13 (0.09)	1.14 (0.95,1.38)	0.14 (0.09)	1.15 (0.95,1.39)	−0.21^*^ (0.10)	0.81^*^ (0.67,0.99)	−0.18 (0.10)	0.83 (0.68,1.01)
Ethnicity								
Muslim	0.26 (0.14)	1.30 (0.98,1.71)	0.25 (0.14)	1.28 (0.98,1.68)	0.45^**^ (0.15)	1.57^**^ (1.15,2.12)	0.29 (0.15)	1.34 (0.99,1.81)
Tamil	0.68^***^ (0.21)	1.97^***^ (1.30,3.00)	0.51 (0.27)	1.66 (0.98,2.81)	0.95^***^ (0.25)	2.58^***^ (1.59,4.21)	1.11^***^ (0.23)	3.04^***^ (1.92,4.81)
Income (SL Rupees)	−0.00^***^ (5.11)	1.00^***^ (1.00,0.99)	−7.91 (5.00)	1.00 (0.99,1.00)	−0.00^***^ (5.19)	1.00^***^ (0.99,1.00)	−0.00^*^ (5.17)	1.00^*^ (0.99,1.00)
Father’s education								
Grade 6-12	0.59^**^ (0.23)	1.80^**^ (1.15,2.83)	0.28 (0.24)	1.33 (0.83,2.13)	0.16 (0.23)	1.18 (0.75,1.85)	(0.01) (0.32)	1.01 (0.55,1.89)
Grade ≤ 5	1.02^***^ (0.27)	2.77^***^ (1.64,4.67)	0.83^**^ (0.29)	2.30^**^ (1.30,4.06)	0.47 (0.27)	1.60 (0.94,2.72)	0.07 (0.36)	1.08 (0.53,1.20)
Mother’s education								
Grade 6-12	0.40^**^ (0.14)	1.49^**^ (1.12,1.98)	0.18 (0.15)	1.19 (0.89,1.61)	−0.05 (0.16)	0.95 (0.69,1.30)	−0.19 (0.23)	0.83 (0.52,1.31)
Grade ≤ 5	0.45 (0.24)	1.57 (0.98,2.51)	−0.26 (0.27)	0.77 (0.45,1.30)	0.52^*^ (0.23)	1.69^*^ (1.07,2.67)	0.19 (0.30)	1.21 (0.67,2.19)

^*^p < 0.05 ^**^p < 0.01 ^***^p < 0.001.

IRR = Incidence Rate Ratio SE = Standard Error 95% CI = 95% Confidence Interval.

Reference categories: Gender- girls, Ethnicity- Sinhalese, Father’s and Mother’s education- > grade 12.

**Table 4:** Associations between selected socio-demographic variables and oral health-related quality of life

**Variables**	**ECOHIS score (child)**				**ECOHIS score (family)**			
	**Unadjusted coefficient (SE)**	**IRR (95% CI)**	**Adjusted coefficient (SE)**	**IRR (95% CI)**	**Unadjusted coefficient (SE)**	**IRR (95% CI)**	**Adjusted coefficients (SE)**	**IRR (95% CI)**
Age	0.02^*^ (0.01)	1.02^*^ (1.00,1.05)	0.02 (0.01)	1.02 (0.99,1.04)	0.00 (0.00)	1.00 (0.99,1.01)	0.00 (0.00)	1.00 (0.99,1.01)
Gender								
Boys	0.06 (0.12)	1.06 (0.84,1.34)	0.12 (0.12)	1.13 (0.89,1.43)	0.00 (0.06)	1.00 (0.88,1.13)	0.03 (0.07)	1.03 (0.91,1.18)
Ethnicity								
Muslim	0.43^*^ (0.21)	1.54^*^ (1.02,2.32)	0.37 (0.22)	1.45 (0.95,2.23)	0.41^***^ (0.12)	1.51^***^ (1.18,1.92)	0.35^**^ (0.13)	1.42^**^ (1.10,1.84)
Tamil	0.76^***^ (0.23)	2.15^***^ (1.36,3.40)	0.73^**^ (0.28)	2.07^**^ (1.19,3.60)	0.12 (0.18)	1.13 (0.79,1.61)	−0.14 (0.15)	0.87 (0.65,1.17)
Income								
(SL Rupees)	−7.49 (6.05)	1.00 (0.99,1.00)	4.47 (6.16)	1.00 (0.99,1.00)	−1.10 (3.15)	1.00 (0.99,1.00)	4.82 (3.26)	1.00 (0.99,1.00)
Father’s education								
Grade 6-12	0.71^**^ (0.25)	2.04^**^ (1.25,3.34)	0.53 (0.29)	1.69 (0.96,2.98)	0.37^***^ (0.06)	1.45^***^ (1.29,1.63)	0.31^**^ (0.11)	1.37^**^ (1.11,1.69)
Grade ≤ 5	0.77^*^ (0.32)	2.15^*^ (1.15,4.05)	0.92^*^ (0.38)	2.52^*^ (1.20,5.31)	0.35^**^ (0.13)	1.42^**^ (1.10,1.82)	0.46^*^ (0.18)	1.59^*^ (1.11,2.27)
Mother’s education								
Grade 6-12	0.38^*^ (0.18)	1.46^*^ (1.02,2.07)	0.13 (0.20)	1.14 (0.77,1.68)	0.21^*^ (0.08)	1.23^*^ (1.04,1.45)	0.15 (0.11)	1.16 (0.94,1.43)
Grade ≤ 5	0.01 (0.44)	1.01 (0.43,2.38)	−1.16^***^ (0.36)	0.31^***^ (0.15,0.64)	0.13 (0.19)	1.14 (0.79,1.65)	−0.31 (0.16)	0.74 (0.53,1.02)

^*^p < 0.05 ^**^p < 0.01 ^***^p < 0.001.

IRR = Incidence Rate Ratio SE = Standard Error 95% CI = 95% Confidence Interval.

Reference categories: Gender- girls, Ethnicity- Sinhalese, Father’s and Mother’s education- > grade 12.

Multivariate analysis showed that caries was significantly higher in children whose fathers had ≤ 5 years of education compared to those whose fathers had >12 years of education (IRR = 2.30; 95% CI: 1.30, 4.06; p < 0.01) while gingival bleeding was significantly higher in Tamil children (IRR = 3.04; 95% CI: 1.92, 4.81; p < 0.001) compared to Sinhala children and in children of low income earners compared to children of higher income earners (IRR = 1.00; 95% CI:0.99,1.00; p < 0.05) (Table 3). Poor OHQoL in children as measured by ECOHIS child scores was associated with Tamil ethnicity (IRR = 2.07; 95% CI: 1.19, 3.60; p < 0.01) and low level of education of father (≤ grade 5: IRR = 2.52; 95% CI: 1.20, 5.31; p < 0.05) (Table 4). However, children whose mothers had ≤ 5 years of education had lower ECOHIS scores, hence better OHQoL than those whose mothers had >12 years of education (IRR = 0.31; 95% CI:0.15,0.64; p < 0.001). Higher ECOHIS family scores (i.e. poor OHQoL at family level) were associated with Muslim ethnicity (IRR = 1.42; 95% CI: 1.10, 1.84; p < 0.01) and lower levels of education of fathers (> grade 12: IRR = 1.37; 95% CI: 1.11, 1.69; p < 0.01) (≤ grade 5: IRR = 1.59; 95% CI: 1.11, 2.27; p < 0.05) (Table 4).

## Discussion

The prevalence of dental caries among children in the present study (72%) is comparable to the prevalence rates reported for children of similar ages from neighbouring and other low- and middle-income countries. For example, Hashim et al. examined dental caries experience and use of dental services among preschool children in Ajman, United Arab Emirates. They found that the prevalence of dental caries among preschool children was 76.1%, [34], while 62% of 3–5 year olds from Uganda had dental caries [19]. In the present study, bleeding gums were present in 47% of the sample. In Bangladesh it was found that gingival bleeding was present among 67% of children aged between 5–15 years [35].

Although in our adjusted model we found that the association between mother’s education and caries levels disappeared, higher caries levels were associated with low educational attainment of the father (Table 3). Moreover, Hashim et al. have shown that poor child oral health is associated with low educational attainment among mothers [34]. Similar findings with regard to the association between poor oral health of the child and parental education have been observed in several other studies. For example, having reviewed findings from several national surveys of children between 2–4 years and 6–8 years, Edelstein reported that children of parents with less than high school education had the highest caries experience compared to children of parents with higher education levels [36]. In another study carried out among Brazilian preschool children, it was found that children who had parents with <8 years of education experienced higher levels of caries than the children of more educated parents [37]. However, contrary to these findings, Schroth et al. found that caregiver’s education was not associated with early childhood caries (ECC) [38], and the authors attributed their finding to the more equal levels of education of the care- givers who participated in that study.

We have found that higher bleeding tendencies are associated with lower income levels (Table 3). Similar findings have been demonstrated in research indicating that low family income is associated with poor oral health outcomes of children. For example, Finlayson et al. found that children under 6 years of age of low income families are more prone to caries [39]. In a study done among Brazilian preschool children, Piovesan et al. found that children of families with low household incomes had higher caries levels than the children of families with higher incomes [40].

Furthermore, we found that higher bleeding tendencies are associated with the Tamil ethnic group (Table 3). This finding demonstrates the existence of ethnic differentials in oral health outcomes. Hashim et al. also found that Emirati children have poorer oral health than children of other ethnicities [34]. Similarly Piovesan et al. found that non-white children have higher caries levels than white children [40]. Tinanoff and Reisine indicate that ethnic minority groups experience poor oral health outcomes due to various factors, such as how health care providers interact with them, accessibility to resources, level of patient’s trust and patient perceptions about the etiology, course and outcomes of disease [41].

The OHQoL of the child is significantly associated with Tamil ethnicity, and low levels of education of the father (≤ grade 5) (Table 4). Piovesan et al. found that low household income is a risk factor for poor OHQoL in children (RR = 1.13) [42]. This finding explains our finding of an association of low education of the father with lower OHQoL of the child. Obviously, if the father of the family has a low education level, the income of the family is reduced.

Piovesan et al. state that children of mothers with low education experience poorer OHQoL (RR = 1.3) [42]. In contrast, our research found that low educational attainment among mothers was associated with better OHQoL. This significant negative association was observed in the lowest (≤ grade 5) education category. Low education level may lead to unemployment [42], which is consistent with our data that show >94% unemployment in the above group. It has been found that children whose mothers are employed are more likely to consume sweetened beverages and less likely to consume fruit and vegetables between meals [43]. Obviously, this may lead to poor oral health and poor OHQoL of the children. Moreover, unemployed mothers have more time to observe and guide their children in tooth brushing, to take their children to government dental clinics which provide free services, and to participate in free workshops and public health events that provide current oral health knowledge than their employed counterparts.

Poor OHQoL of the family is significantly associated with Muslim ethnicity and lowest level of father’s education (≤ grade 5) (Table 4). According to our demographic data, compared to Sinhala families, in Muslim families there is a relatively large number of siblings. With this high number, the attention given to each child and other family members may not be adequate. Oral health is no an exception to this phenomenon. We also found that 32% of the Muslim families belonged to the lowest income level, whereas only 13% of Sinhala families belonged in that category. Therefore, as a result of increased family size and lower economic level, oral health-related quality of life of the family may deteriorate. A study carried out in Sri Lanka has demonstrated consistent results, and the authors indicate that children who had ≥2 siblings had significantly more caries than children of families with ≤2 siblings [27]. On the other hand, the lower education level of the father may adversely affect OHQoL of the family, for the reasons mentioned in the previous paragraph.

Comparable studies undertaken in countries with very disadvantaged population sub-groups have also demonstrated similar results with regard to oral health outcomes such as dental caries and gingival bleeding [17,34,35]. Hence, there is the possibility of generalizing of our findings to other areas in Sri Lanka and other countries, especially those with similar socioeconomic backgrounds.

There are some methodological limitations of this study. For example, there may have been situations where the respondents were reluctant to divulge information. When administering the questionnaires to Tamil speaking respondents, the assistance of a competent interpreter was obtained. However, it is possible that certain questions may have been misinterpreted.

## Conclusions

Father’s education is an important factor in determining dental caries and OHQoL among preschool children. Members of ethnic minorities carry the greatest burden of poor oral health outcomes, with Tamils having the highest level of bleeding gums and poorest child OHQoL and Muslims having the poorest OHQoL at the family level. Therefore, father’s education level and ethnicity are the key dimensions to consider when planning and providing oral health services to preschool children in this area.

## Abbreviations

Dmfs: Decayed missing and filled surfaces; DSD: Divisional secretariat division; ECC: Early childhood caries; ECOHIS: Early childhood oral health impact scale; OHQoL: Oral health-related quality of life.

## Competing interests

The authors declare that they have no competing interests.

## Authors’ contributions

VN and LE designed the study. Data collection, including oral examination of the children and administering the questionnaires, was done by VN. AR did the data analysis. All authors were involved in interpreting the data, while VN and LE drafted the manuscript. AR and BO revised the draft critically. All authors have given their consent to the final draft.

## Authors’ information

VN (BDS, MSc) – Visiting Academic, Global Health and Society Unit, Monash University; Senior Registrar (Community Dentistry), Ministry of Healthcare and Nutrition, Sri Lanka.

AR (PhD, MPH, MPHAA) – ARC Future Fellow and Director Migration, Social Disadvantage, and Health Programs, Department of Epidemiology & Preventive Medicine, Monash University; Senior Fellow- Nutrition, Centre for International Health, Burnet Institute.

BO- (PhD) - Professor of International Public Health; Associate Dean (Global Health and International Campuses), Monash University; Director, Global Health and Society Unit, Monash University.

LE (BDS, PhD) - Professor in Community Dentistry, Department of Community Dental Health, Faculty of Dental Sciences, University of Peradeniya, Sri Lanka.
